# Atypical Diabetic Foot Ulcer Keratinocyte Protein Signaling Correlates with Impaired Wound Healing

**DOI:** 10.1155/2016/1586927

**Published:** 2016-10-20

**Authors:** Glenn D. Hoke, Corrine Ramos, Nicholas N. Hoke, Mary C. Crossland, Lisa G. Shawler, Joseph V. Boykin

**Affiliations:** ^1^Theranostics Health, Inc., Gaithersburg, MD 20877, USA; ^2^HCA Chippenham Medical Center, Wound Healing Center, Richmond, VA 23235, USA; ^3^HCA Retreat Doctors' Hospital, Wound Healing Center, Richmond, VA 23220, USA

## Abstract

Diabetes mellitus is associated with chronic diabetic foot ulcers (DFUs) and wound infections often resulting in lower extremity amputations. The protein signaling architecture of the mechanisms responsible for impaired DFU healing has not been characterized. In this preliminary clinical study, the intracellular levels of proteins involved in signal transduction networks relevant to wound healing were non-biasedly measured using reverse-phase protein arrays (RPPA) in keratinocytes isolated from DFU wound biopsies. RPPA allows for the simultaneous documentation and assessment of the signaling pathways active in each DFU. Thus, RPPA provides for the accurate mapping of wound healing pathways associated with apoptosis, proliferation, senescence, survival, and angiogenesis. From the study data, we have identified potential diagnostic, or predictive, biomarkers for DFU wound healing derived from the ratios of quantified signaling protein expressions within interconnected pathways. These biomarkers may allow physicians to personalize therapeutic strategies for DFU management on an individual basis based upon the signaling architecture present in each wound. Additionally, we have identified altered, interconnected signaling pathways within DFU keratinocytes that may help guide the development of therapeutics to modulate these dysregulated pathways, many of which parallel the therapeutic targets which are the hallmarks of molecular therapies for treating cancer.

## 1. Introduction

Chronic wound complications are a growing concern worldwide and the impact is a threat to public health and the economy [1]. The growing global prevalence of diabetes affects all populations and is associated with obesity, impaired wound healing, and chronic DFU formation. Worldwide, the number of people with diabetes is projected to rise from 171 million in 2000 to 366 million in 2030 with this “diabetic epidemic” continuing even if levels of obesity remain constant [2]. There are over 30 million people in the US with diabetes mellitus (9.3% of US population) and the estimated direct costs exceed $170 billion annually [3]. Diabetic wound complications include progressive tissue loss, soft tissue and boney wound infections, accelerated cardiovascular disease, lower extremity amputations, and patient mortality [4]. A common complication for patients with diabetes, the lifetime risk for lower extremity ulceration, is as high as 25%, with over 7% of individuals with diabetic neuropathic foot ulcers progressing to amputation [5]. Additionally, for public and private payers, the financial burden of treating DFU complications is estimated at $13 billion above the costs of diabetes itself [6]. Currently, no methods are available to identify those diabetic patients with lower extremity wounds that will demonstrate normal wound healing and recovery as compared to those whose wounds recur and worsen. There is an unmet need for novel research and technological applications to identify the cellular mechanisms responsible for impaired diabetes wound healing and its complications and to guide new therapeutic development.

The paradigm for wound healing is divided into four overlapping stages: hemostasis, inflammation, proliferation, and remodeling. This process requires a complex coordination of key molecular, cellular, and physiologic events by facilitative signaling between hematopoietic, immunologic, and resident skin cells [7]. Increased expression of the tumor suppressor transcription factor p53 and ischemia-induced apoptosis may result in senescence and the inhibition of signaling pathways driving inflammation or cell survival, depending upon which diabetic wound healing models are evaluated [8–10]. Other proteins identified in healing models, like the hypoxia-inducible factor-1 (HIF-1) and HIF-1*α* proteins (which modulate angiogenesis, cell proliferation, and wound healing, as well as cancer invasion/metastasis), may be altered in the diabetic environment, contributing to impaired wound healing [11–13]. The dysregulation of signaling pathways in several models used to evaluate diabetic wound healing has been correlated with alterations in the levels of micro-RNAs (miRNAs), which comprise families of highly conserved small noncoding RNA molecules, that bind to and coordinately regulate signaling pathways by interfering with mRNA translation of associated proteins [14]. These observations suggest that a comprehensive assessment of protein signaling cascades related to proliferation, migration, inflammation, and apoptosis/senescence in diabetic wounds might identify novel therapeutic strategies and diagnostics for identifying alterations in signaling activity that define healing from nonhealing DFUs. The scope of this preliminary study was to perform a correlative assessment of critical wound repair signaling pathway proteins in DFUs.

## 2. Materials and Methods

All clinical investigation was performed at the HCA Retreat Doctors' Hospital Wound Healing Center, Richmond, VA. The study protocol and consent forms were approved by the Western Institutional Review Board (WIRB) (Study number 1134749, title: A Single Center Quantitative Proteomic Assessment of Impaired Wound Healing Following Diabetic Foot Ulcer Development). The study was conducted in accordance with the Declaration of Helsinki (1964) and the Belmont Report (1979) as related to the ethical principles and guidelines for research involving human subjects. The reported study activities were conducted with the human subjects' understanding and prospective written informed consent.

### 2.1. Study Subjects and Tissue Specimen Procurement

DFU subjects were adults with chronic, full-thickness, neuropathic foot ulcers selected from the patient population of the HCA Retreat Doctors' Hospital Wound Healing Center Clinic (WHCC). Control study subjects were nondiabetic adults evaluated by the study principal investigator in the WHCC who had volunteered to submit plantar foot tissue specimens for control tissue analysis. During this study, full-thickness plantar foot skin samples were obtained using a three-millimeter punch biopsy technique and submitted for RPPA analysis. Following the recruitment of twenty-five DFU subjects and five control subjects for the study, eighteen (*n* = 18) DFU subjects' and three control subjects' (*n* = 3) tissue biopsy samples were found to be suitable for RPPA analysis. For DFU subjects, two adjacent three-millimeter punch specimens were obtained from the viable ulcer margin immediately following clinical debridement of the wound. Two adjacent three-millimeter punch specimens were also obtained from control subject's plantar foot areas. For tissue biopsies, all study subjects were allowed to receive a suitable volume of injectable local anesthetic for tissue retrieval, if needed. The punch biopsy sites were closed by use of nonabsorbable sutures and dressed with occlusive foam or silicon bandages. All subject tissue specimens were immediately placed in formalin to preserve morphology and phosphoprotein levels in tissues. Control subjects were followed up in the WHC until wound closure (about 3 weeks). DFU subject's tissue sample sites were dressed appropriately for their designated care plan and for offloading. DFU study participants continued with normally scheduled wound evaluations in the WHCC as routinely coordinated by the medical and nursing staff. A twelve-week postdebridement observation period was scheduled for all DFU study subjects to assess wound healing and study subject compliance and for evaluation of offloading methods assigned for DFU subject care. At the end of the twelve-week observation period, three (*n* = 3) DFU subjects were observed with complete healing of the study ulcer.

In the DFU study group, there were seventeen subjects with type II diabetes and one with type I diabetes, fifteen male and three female subjects. The average age (mean value) of the DFU subjects was 60.3 years (ranging from 48 to 75; median age = 60). The average age of the DFU study ulcer was nineteen (19) months, ranging from 2 to 120 months (median ulcer age = 11 months). The wound age in months (±STDev) for the healed and unheald DFUs was as follows: mean ulcer age – healed DFUs = 18.0 months (±16.1) versus unhealed DFUs = 19.2 months (±19.2). The mean DFU wound sizes were measured by planometric methods and were 7.5 cm^2^ (±13.2 cm) for unhealed DFUs versus 1.6 cm (±1.0 cm) for healed DFUs. Healed and unhealed DFU wound sizes were not significantly different. The control study group (*n* = 3) was comprised of three healthy adult males with an average age of 23 years.

DFU subject wound care consisted of moist to dry dressings, foams, and alginates with and without nanocrystalline silver, collagenase, antibiotic ointments, or hydrogels with or without nanocrystalline silver. No study subjects received allograft, xenograft, living, single or bilayered human cell based dressings (e.g., Dermagraft™ or Apligraf™), or adjunctive hyperbaric oxygen therapy (HBOT) during or within six months of this study.

DFU study subjects had been taking medications for glycemic control prior to tissue specimen retrieval. These medications were not changed during the study observation period and are listed in Table 1. No statistical correlations could be made between subjects using Metformin and healed or unhealed status (data not shown) or the ulcer wound size.

### 2.2. Tissue Specimen Preparation and Reverse-Phase Proteomic Array Investigation

Biopsies preserved in formalin were sectioned and the keratinocytes were isolated using macrodissection. Whole cell lysates are prepared by boiling 10 minutes in a sodium dodecyl sulfate-based lysing solution to produce a denatured lysate that is robotically printed (Aushon 2470 Arrayer) on slides coated with nitrocellulose. Multiple, replicate slides are printed and each individual replicate is reacted with a single primary antibody directed against the epitope of interest. As a calibrated assay, each array consists of the following:

(a) Experimental subject samples are printed, *n* = 4, at two different dilutions.
(b) High and low positive and negative controls are printed, *n* = 2, at four different dilutions.
(c) A calibration standard curve for each analyte is measured, consisting of 6 to 10 concentrations of each analyte contained in a constant protein background spanning the dynamic range of the analyte of interest.

Printed arrays are stained for total protein by staining with Sypro® Ruby Protein Blot Stain (Molecular Probes, Eugene, OR). The Sypro Ruby Protein stain signal serves as a measure of the amount of protein deposited within each defined spot on the slide. The stain binds to proteins without bias and does not interfere with subsequent antibody binding.

Slides undergo a blocking procedure using a casein-based solution to block nonantigenic sites and arrays are then probed with the primary antibody. All primary antibodies were prevalidated for protein specificity using Western blotting in the presence and absence of exogenously added peptides representing each protein's unique epitope. Keratinocyte lysates were printed in dilutions to ensure that samples were in the linear range for each individual protein's RPPA assay based upon the performance of the calibrators. A DAKOCytomation Autostainer is used to perform the fluid handling (antibody depositions, washing, and amplification) for RPPA. Reaction with a secondary antibody, that recognizes a constant region across all primary antibodies, provides a biotin-binding site for a streptavidin-based, tyramide-signal amplification that yields detection sensitivities to fewer than 1,000 to 5,000 molecules per spot with good linearity (correlation coefficient or *R* ^2^ = 0.990–0.999) and interexperiment precision (*R* ^2^ = 0.973).

Dual laser scanning (GenePix, Molecular Devices, Inc.) is performed to obtain the sample's fluorescence intensity value. Background values are calculated and subtracted to generate each spot's fluorescence value, which is normalized to the total protein to generate a Normalized Fluorescence Intensity (NFI) value. NFIs are fit to analyte-specific, nonparametrical calibration curves to generate final intensity values. The median of replicates values is reported. Between-run and within-run analytical precision is between 5 and 10% CV (coefficient of variation).

## 3. Statistical Analysis

Summary statistics, such as mean (±SEM) for quantitative measures between categories and ratios, were generated. Statistical analyses were performed using XLSTAT™ and visual analytic analysis using TIBCO® Spotfire®. The primary relationships of interest, the correlation between the control subjects (*n* = 3) and the DFU subjects (*n* = 18), and the correlation between healed subjects (*n* = 3) and unhealed subjects (*n* = 15) were studied using nonparametric Wilcoxon Rank-Sum test. Statistical significance refers to a *p* value of <0.05 and <0.01. Means of the normalized values for each protein were subject to conditional formatting with the highest value assigned a red color, middle values a white color, and the lowest values a blue color and intermediate values colored with the appropriate shades depending upon where they fall within the range for that protein. The heat map was combined with hierarchical clustering to arrange subjects/markers in a hierarchy based on the similarity between subject profiles. The result of a hierarchical clustering calculation is displayed in the heat map as a dendrogram, which is represented by a tree structure of the hierarchy.

## 4. Results

**Figure 1:**
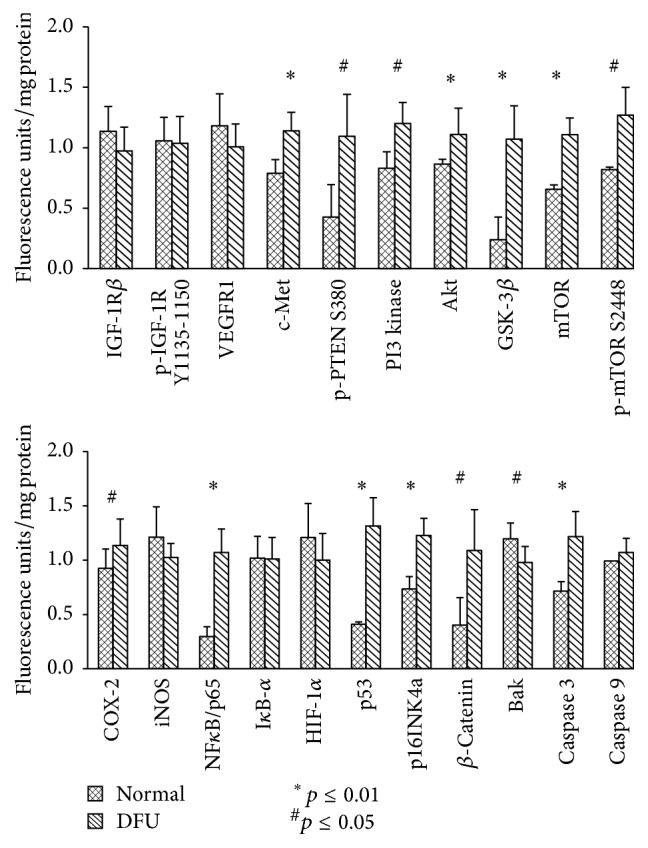
Mean, STDev, and nonparametric Mann–Whitney* U* test results for 21 proteins levels measured in normal and DFU keratinocytes. Mean, STDev, and Mann–Whitney* U* test significance (^*∗*^ *p* ≤ 0.01 or ^#^ *p* ≤ 0.05) for 21 proteins assessed by RPPA in DFUs (*n* = 18) and normal keratinocytes (*n* = 3). Analytes: receptor kinases: IGF-1R*β*, insulin growth factor-1 receptor beta; p-IGF-1R*β* Y1135-1150, insulin growth factor-1 receptor beta (phosphor-tyrosine 1135–1150); VEGFR1, vascular endothelial growth factor receptor-1; and c-MET, hepatocyte growth factor receptor (HGFR); the PI3 kinase/AKT pathway, p-PTEN S380, phosphatase tensin homolog (phospho-serine 380); PI3 kinase, phosphoinosityl-3-kinase; Akt, Akt1/protein kinase B; GSK-3*β*, glycogen synthase kinase-3*β*; mTOR, mammalian target of rapamycin; and p-mTOR, mammalian target of rapamycin S2448 (phosphor-serine 2448); proinflammatory proteins: COX-2, cyclooxygenase-2, and iNOS, inducible nitric oxide synthase; survival pathways: NF*κ*B/p65, nuclear factor kappa B/p65; I*κ*B-*α*, NF*κ*B inhibitor-alpha; HIF-1*α*, hypoxia-inducible factor-1 alpha; p53, tumor suppressor transcription factor, p16INK4a, cyclin-dependent kinase inhibitor/regulator of senescence; and *β*-catenin, catenin beta-1; and apoptosis pathways: Bak, BCL2-antagonist/killer 1; Caspase 3, cysteine-aspartic protease 3; and Caspase 9, cysteine-aspartic protease 9. ^*∗*^ *p* value less than 0.01; ^#^ *p* value less than 0.05 comparing the normal to DFU values.

Comparative, quantitative proteomic assessment of selected signaling proteins of DFU (*n* = 18) and nondiabetic (normal) plantar skin (*n* = 3) was performed on keratinocytes isolated from three-millimeter punch biopsies. During a 12-week postdebridement observation period, three (*n* = 3) DFU subjects demonstrated complete healing of their study ulcers. RPPA quantitatively measured the expression levels and activation (phosphorylation) status of proteins in signaling pathways relevant to wound healing. RPPA allowed for the simultaneous assessment from all biopsies, providing a high-dimensional “snapshot” of signaling activity in DFU keratinocytes [15]. Twenty-one signaling pathway proteins, spanning multiple pathways, were assessed in normal and DFU keratinocytes. Significant differences were observed in multiple signaling proteins following nonparametric Mann–Whitney* U* test comparison of normal and DFU keratinocytes (Figure 1). These elevated proteins in DFUs are known to be dysregulated to some degree in most cancer types, including breast, colon, lung, pancreatic, ovarian, and hepatic tumors [16–19]. The proteins exhibiting significantly (*p* < 0.01) increased levels in nonhealing DFU keratinocytes compared to normal tissue included members of the PI3K/AKT signaling pathway (p-PTEN S380, PI3 kinase, AKT, GSK-3*β*, and p-mTOR S2448); the NF*κ*B pathway (NF*κ*B/p65 and p53); the *β*-catenin pathway (GSK-3*β*, p16INK4a, and Caspase 3). Total mTOR and COX-2 were also elevated (*p* < 0.05) in nonhealing DFUs.

**Figure 2:**
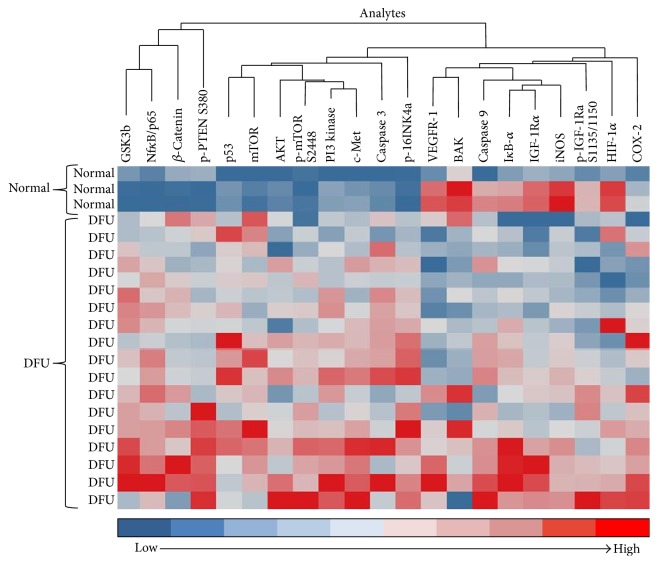
Unsupervised clustering of normal and DFU subjects based upon analyte levels. Unsupervised clustering of normal (*n* = 3) and DFU (*n* = 18) subjects with the dendrogram scaled to represent the distance between each branch. Normalized signal intensities (log⁡2 transformed and row adjustment) are visualized as a color spectrum. Blue color indicates low expression and red color indicates high expression of the detected markers.

The heat map (Figure 2) shows the significant dysregulated signaling pathways between keratinocytes isolated from normal skin and wound margins of DFU subjects. Normal (subjects 19, 20, and 21) and DFU subjects are separated using unsupervised hierarchical clustering. Based upon the signaling architecture of the two populations, DFU subjects significantly exhibit higher levels of multiple proteins across the PI3 kinase/Akt pathway, the survival pathways (NF*κ*B/p65 and *β*-catenin), and the cell surface receptor, c-MET, a target of the TCF/*β*-catenin transcription complex (Figure 2). Thus, RPPA analysis identifies an activity relationship that corresponds to major components of wound healing found in the literature.

**Figure 3:**
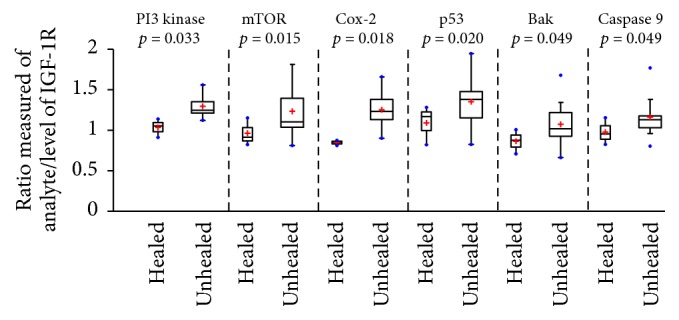
Boxplots showing significantly increased levels of downstream proteins compared to levels of IGF-1R in healed and unhealed DFU keratinocytes. Boxplots showing the calculated ratios for the level of each analyte/level of IGF-1R for healed (*n* = 3) and unhealed (*n* = 15) DFU subjects. These boxplots demonstrate that the unhealed subjects have significantly (*p* ≤ 0.05) elevated ratios of PI3 kinase, mTOR, Cox2, p53, Bak, and Caspase 9 compared to healed subjects. Not only is the center higher for unhealed subjects, but the quantitative independent two-samples* t*-test analysis suggests that the 2-population means differ beyond random variation.

The level of expression (and activation) of IGF-1R, a cell surface receptor involved in insulin and IGF-1 signaling [20, 21], was not significantly different between normal keratinocytes and those from DFUs (healed or unhealed). To assess correlative alterations in downstream signaling, a ratio of the downstream proteins to IGF-1R was calculated in an attempt to identify perturbations to signaling that may distinguish healing from nonhealing DFUs (Figure 3). There were several proteins whose ratios of expression levels were significantly elevated in nonhealing DFU, including PI3 kinase and mTOR, members of the Akt signaling pathway that facilitates cell cycle progression and inhibits apoptotic events; p53, a regulator of cell cycle arrest, apoptosis, and senescence; Caspase 9, indicative of intrinsic apoptosis; Bak, the proapoptotic protein that is increased in response to p53 and is activated by Caspase 9; and COX-2, whose transcription and stabilization may be modulated by Akt or NF*κ*B/p65 signaling, indicative of a proinflammatory state.

## 5. Discussion

Diabetes represents a group of human diseases characterized by prolonged, elevated levels of blood glucose resulting from insulin deficiency or impaired responsiveness of insulin-target cells to circulating insulin [22]. In target tissues, the insulin receptor (IR) and the insulin-like growth factor-1 receptor (IGF-1R) respond to ligand binding by activating downstream signaling cascades [23, 24]. Increased signaling through IR and IGF-1R, leading to activation of PI3K and MAPK kinase pathways, is associated with increased proliferation in cancer [25, 26].

**Figure 4:**
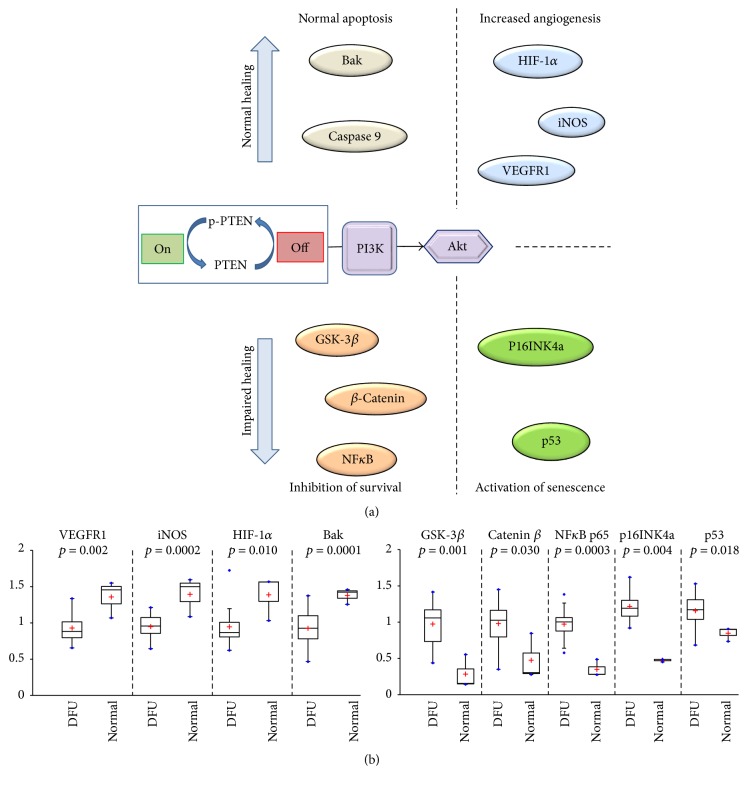
Schematic representation of the signaling dysregulation in DFUs compared to normal tissues and boxplots of those analytes significantly altered between the two tissues. (a) Simplified scheme of the dynamic Akt switch between DFU and normal tissues. Increased levels of PTEN-mediated, PI3 kinase-induced activation of Akt promote competing mechanisms of normal or impaired healing. (b) The calculated ratios for significantly modulated analyte/Akt ratios (boxplots) for DFU (*n* = 18) and normal (*n* = 3) study subjects representative of (1) normal apoptosis (Bak and Caspase-9) and increased angiogenesis (iNOS and VEGFR1) for the normal wound healing response (depicted in (a)) and (2) inhibition of survival (GSK-3*β*, *β*-Catenin, and NF*κ*B) and activation of senescence (p53 and p16INK4a) for the impaired wound healing response (depicted in (a)). These groups may represent potential targets for the development of therapies for the treatment, or prevention, of DFU wounds.

In our analysis, there were no significant differences between normal, healing DFU, and nonhealing DFU levels for total or phosphorylated IGF-1R, suggesting that increased IGF-1R signaling in DFUs was not inhibiting wound healing. However, there was a significant increase in PI3 kinase/mTOR signaling in DFUs, which has been reported to increase the migration of keratinocytes and promote proliferation and reepithelialization in wounds [27, 28]. In murine studies to determine the expression levels of miRNA during the various phases of wound healing, the downregulation of the levels of miRNA-99 family sequences has been shown to significantly increase the mRNA levels for members of the PI3K/AKT/mTOR pathway and slightly (insignificantly) increase the level of IGF-1R expression [29]. The miRNA-99 family's modulation of the PI3K/mTOR signaling pathway and IGF-1R signaling have been observed in a number of cancers, supporting their role in modulating proliferation, migration, and apoptosis [30–33]. The effects of reduced levels of miRNA-directed increases in the expression of proteins in the PI3K/mTOR pathway are to increase the proliferation and migration of keratinocytes, while decreasing apoptosis. In our assessment of human healing in normal and diabetic subjects, the observed significant increased expression levels of PI3K and mTOR proteins in unhealed DFU keratinocytes (Figure 3) may support a reduction of miRNA targeting of these proteins involved in the healing response in diabetic wounds. Examination of the roles that miRNAs exert in regulating signaling activity in diabetic wound healing in murine models has also identified miRNA-146a, which is decreased in diabetic wounds [34, 35]. miRNA-146a has been reported to act as an inhibitor of NF*κ*B signaling, leading to an increase in the beneficial proinflammatory phase of wound healing [36]. Decreased miRNA-146a levels result in increased proinflammatory signaling through the increased expression of IL-1 receptor-associated kinase 1 (IRAK1) and TNF receptor-associated factor 6 (TRAF6), target mRNAs of miRNA-146a [35]. With decreased miRNA-146a, there was an associated increase in IRAK1 and TRAF6 protein levels that correlated with increased NF*κ*B levels leading to increased proinflammatory signaling. In diabetic wounds, a persistent inflammatory phase of the healing process is evident [37] and is a contributing factor to reduced healing [38]. We also observed increased NF*κ*B levels in both healing and nonhealing diabetic ulcers (Figure 4(b)); elevated levels of NF*κ*B are a biological response to hyperglycemia and result in inhibition of leukocyte migration [39] and proliferation [40]. Our data may be confirmatory that the influence of miRNAs, like miRNA-146a, that modulate the inflammatory response in healing wounds was not significantly perturbed in those DFUs that do not heal. Thus, the regulation of keratinocytes by miRNAs appears to be functioning characteristically in both the healing and nonhealing diabetic ulcer. There appear to be other factors in the nonhealing DFU, as there is growing evidence of the roles played by other miRNA families (e.g., miR-191 and miR-200b) that are interfering with the coordinated processes involved in wound repair with diabetes [41]. An assessment of the miRNA levels in the context of activated or inhibited keratinocyte signaling pathways using RPPA would be useful to determine the critical alterations that affect DFU wound healing.

Expression of c-Met was significantly increased in DFUs compared to normal keratinocytes, and c-Met has been identified as critical for wound healing [42]. The observed increased levels of c-Met may also be responsible for the observed increased PI3 kinase/Akt signaling in DFU. The IGF-1R and IR receptors are known to form heterodimer pairs with c-Met, mediating transactivation of c-Met in the absence of its ligand, HGF (hepatocyte growth factor), thereby potentiating unregulated signaling in multiple pathways (DFU levels compared to normal, Figure 2), including regulation of PI3 kinase/Akt, GSK3*β*, and thereby *β*-catenin [43, 44]. Activated c-Met (in response to HGF) is known to activate *β*-catenin signaling [45, 46]. In addition, activated c-Met and IGF-1R have been reported to drive PI3 kinase signaling in colorectal cancers [46]. The signaling architecture observed in this study suggests that in DFUs patterns of cross talk exist between pathways involved in apoptosis (p53, caspases), proliferation and migration (PI3 kinase/Akt/mTOR), inflammation (Cox-2, NF*κ*B), and senescence (p53, p16INK4a).

RPPA identified active signaling pathways in DFUs which may help identify points of therapeutic intervention to treat nonhealing DFUs (Figure 3). Observed differences between healing and nonhealing DFUs suggest an increased level of proapoptotic proteins and a potential role for c-Met/IGF-1R heterodimer signaling through the PI3 kinase/Akt pathway as an important driver in the nonhealing wound. In the absence of altered levels of IGF-1R or its activation by phosphorylation, a significant modulation of pathways related to increased PI3 kinase/AKT signaling coupled with increased inflammatory and apoptotic signaling occurs, perhaps in response to c-Met activation.

The nonhealing DFU is a disease of dysregulated signal transduction potentially influenced by somatic mutations and miRNA expression levels. Altered signaling due to somatic mutations can interfere with mRNA or miRNA transcription resulting in altered levels of proteins leading to modulation of protein function. These mutations change signaling efficiency, altering the control of transcription factors that modulate the intercellular levels of key proteins or the regulated patterns of posttranslational modifications (PTM). Indeed, similar dysregulation of Akt, a major signaling node in cells regulating multiple signaling pathways associated with the development of diabetes, cancer, and cardiovascular disease, has been documented [47]. Unlike the impact of increased PI3 kinase/Akt signaling in the development of cancers, in wound healing, PI3 kinase/Akt activates apoptotic and angiogenesis pathways [48]. An interesting observation in DFUs is the PTEN-inactivating phosphorylation (p-PTEN) at S380, resulting in increased PI3 kinase/Akt signaling which may trigger senescence in addition to survival (Figure 4(a)). Thus, the quantitative measurement of PTEN/PI3 kinase/Akt imbalances may provide a useful set of biomarkers that can be exploited for use as a diagnostic to personalize therapy, or suggestive of therapeutic targets for future development, thereby, contributing to increased healing in DFUs (Figure 4(b)).

Many of the active signaling pathways we observed in DFUs are also dysregulated in cancers, suggesting that drugs developed for one disease may have utility in treating the other. For example, the diabetes drug Metformin, an activator of AMPK-directed inhibition of Akt/mTOR-mediated proliferation and Cyclin D1 synthesis, reduces the incidence and progression of some cancers [25]. In diabetics, Metformin may be associated with larger ulcerations and inhibition of keratinocyte proliferation, but these DFUs do not progress to amputation [49]. These results suggest that activation of survival inhibition and senescence signaling pathways, like p53 and *β*-catenin, may contribute to nonhealing DFUs. There may be opportunities to treat nonhealing DFUs using topical applications of therapies targeting proteins in these pathways or for the development of diagnostic tests to guide therapeutic decisions when treating DFUs.

## 6. Conclusions

Using RPPA to analyze the signaling architecture of keratinocytes isolated from healing and nonhealing DFUs and normal tissues, we have identified an interconnection of multiple signaling pathways relevant to wound healing that may result from an increased signaling through Akt, which is further mediated by the stabilization of PTEN through phosphorylation at serine 380, which reduces PTEN biological activity. Upon wounding, downregulation of the miR-99 family members leads to the upregulation/activation of AKT/mTOR signaling pathway, which in turn activates cell proliferation and migration, and facilitates wound closure. However, the increased level of p-PTEN observed in our study may suggest that the control of Akt/FOXO-1 signaling, leading to decreased proliferation and migration and increased apoptosis, may counteract the effects of altered levels of miRNA99-responsive protein members. For example, in cervical cancers and in breast cancer cells, the levels of miRNA181a, or miRNA29a, modulate the expression levels of PTEN, resulting in decreased rates of proliferation and migration or increased apoptosis [50, 51]. Phosphorylated PTEN reduces the PTEN-mediated inhibitory effect on signaling through the PI3K/Akt pathway. Thus, increased signaling through PI3K/Akt modulates the activity of multiple pathways involved in apoptosis, cell survival, senescence, angiogenesis, and proliferation. By measuring the activation of key proteins in these pathways, it may be possible to predict which DFUs will heal and those that will not. In addition, this work identified several nodes along these pathways that may be appropriate therapeutic targets for treating the nonhealing ulcers.

Normal wound healing of the epidermis is dependent upon the balanced coordination of cellular (keratinocyte) proliferation and differentiation that may be differentially regulated by the presence of miRNAs during the phases of wound healing. In this case, the sequenced proliferation of mitotically active keratinocytes and differentiation of postmitotic cells require the regulation and modulation of multiple signaling molecules and pathways [52]. IGF-1 is one of the major regulators of cellular proliferation and differentiation [53]. Insulin and insulin-like growth factor-1 (IGF-1) signaling, by IGF-1R, are differentially involved in keratinocyte differentiation, promoting the inhibition of normal keratinocyte differentiation, suggesting that abnormal insulin signaling, as what occurs in diabetes, may lead to skin pathology [54]. Increasing levels of IGF-1 or IGF-1R are also associated with increased cell proliferation, skin hyperplasia, and tumorigenesis [55]. In our analysis, there were no significant differences between normal, healing DFU, or nonhealing DFU levels for total or phosphorylated IGF-1R suggesting that increased IGF-1R signaling in DFUs was not inhibiting wound healing.

Also in our study, there was a significant increase in PI3K/mTOR signaling in healing and nonhealing DFUs. PI3K and mTOR have been reported to increase keratinocyte migration and promote proliferation and wound epithelialization [27, 28]. Alternatively, these same signaling molecules have been demonstrated to regulate keratinocyte differentiation and participate in the mediation of the IGF-1R inhibitory signaling for keratinocyte differentiation [56]. Alterations in the signaling architecture of keratinocytes, particularly those that modulate proliferation, migration, and apoptosis, are under the influence of many factors, including the constellation of miRNAs that are present in the cells.

Our analysis of the signaling protein expression ratios of PI3K/IGF-1R and mTOR/IGF-1R (as well as p53/IGF-1R, COX-2/IGF-1R, Bak/IGF-1R, and Caspase-9/IGF-1R) documents a correlation of these values with healing outcomes of DFUs suggesting that there may be threshold values of these signaling relationships, for example, through Akt, a master regulator of multiple signaling pathways, engaged in the regulation of keratinocyte proliferation, differentiation, and migration, that are closely associated with successful wound repair. While there are alternative explanations for these relationships that require further evaluation, we suggest that these expression ratios may function as predictive or diagnostic biomarkers for DFU wound healing (see Figure 3). We suggest that there may be substantial clinical value for the use of the proteomic biomarkers described in this study that would include the development and promotion of more effective and efficient clinical DFU management and as a research platform for the discovery and development of novel therapeutic solutions for the pharmaceutical treatment of dysregulated signaling networks for the reduction, or prevention, of the complications of DFUs. Follow-up studies using RPPA are planned to expand upon the number of signaling proteins measured in normal and DFU tissues. In addition to the pathways measured herein, additional pathways, including the MAPkinase (Ras/Raf/Mek/Erk), p38-MAPK/SAPK, and Akt-dependent pathways, will be included.

## Figures and Tables

**Table 1:** Diabetic medications taken during the study. DFU study subjects listed by number with their prestudy medications for glycemic control. Healed indicates subjects with healed study DFU wounds at the completion of the twelve-week observation period. TIDM indicates study subject with type I DM; all other subjects are with type II DM.

Patient number	Prior therapy	DFU status
1	Tradjenta (linagliptin)	Nonhealing
2	Lantus/Humalog Insulin	Nonhealing
3	Glimepiride	Nonhealing
4	Glipizide; Metformin	Healed
5	Lantus/Humalog Insulin	Nonhealing
6	Humalog/Humulin N Insulin	Nonhealing
7	Humalog/Humulin N Insulin (TIDM)	Nonhealing
8	Humulin 70/30 Insulin	Nonhealing
9	Glipizide/Novolog and Lantus Insulin	Healed
10	Glimepiride/Victoza and Invokana Insulin	Nonhealing
11	Glyburide/Lantus and Humalog Insulin; Metformin	Healed
12	Glimepiride; Metformin	Nonhealing
13	Humulin 70/30 Insulin	Nonhealing
14	Lantus Insulin	Nonhealing
15	Novolog and Lantus Insulin	Nonhealing
16	Lantus and Novolog Insulin, Metformin	Nonhealing
17	Novolin N and Novolin R Insulin; Actos	Nonhealing
18	Glipizide, Metformin, Januvia	Nonhealing
